# Oncologic outcomes of single-incision laparoscopic surgery versus conventional laparoscopic surgery for colorectal cancer (CSILS): study protocol for a multicentre, prospective, open-label, noninferiority, randomized controlled trial

**DOI:** 10.1186/s12885-022-09821-9

**Published:** 2022-07-07

**Authors:** Zijia Song, Kun Liu, Tao Zhang, Bingshun Wang, Yiqing Shi, Yimei Jiang, Changgang Wang, Xianze Chen, Xiaopin Ji, Ren Zhao

**Affiliations:** 1 Department of General Surgery, Ruijin Hospital, Shanghai Jiao Tong University School of Medicine, No. 197, Ruijin Er Road, Huangpu District, Shanghai, 200025 China; 2 Department of Biostatistics and Clinical Research Center, Shanghai Jiao Tong University School of Medicine, No. 227, South Chongqing Road, Huangpu District, Shanghai, 200025 China

**Keywords:** Study protocol, Colorectal cancer, Oncologic outcomes, Single-incision, Laparoscopic surgery, Randomized controlled trial

## Abstract

**Background:**

In most previous studies, single-incision laparoscopic surgery (SILS) for colorectal cancer (CRC) was feasible and safe in the short term. However, long-term oncologic outcomes remain uncertain, as only a few studies contained long-term survival data. SILS for CRC is still in the early stages of research. Further studies, particularly large-scale, prospective randomized controlled trials, are necessary to assess the value of SILS for CRC.

**Methods:**

This study is a prospective, multicentre, open-label, noninferiority, parallel-group randomized controlled trial that investigates the long-term oncologic outcomes of SILS compared to conventional laparoscopic surgery (CLS) for CRC. A total of 710 eligible patients will be randomly assigned to the SILS group or the CLS group at a 1:1 ratio using a central, dynamic, and stratified block randomization method. Patients with ages ranging from 18 to 85 years old, of both sexes, with CRC above the peritoneal reflection diagnosed as cT1-4aN0-2M0 and a tumour size no larger than 5 cm will be considered for the study. The primary endpoint is 3-year disease-free survival (DFS). The secondary endpoints include: intraoperative outcomes, postoperative recovery, postoperative pain assessment, pathological outcomes, early morbidity and mortality rate, cosmetic effects, quality of life, 3-year overall survival (OS), incidence of incisional hernia, 5-year DFS and 5-year OS. The first two follow-up visits will be scheduled at one month and three months postoperatively, then every three months for the first two years and every six months for the next three years.

**Discussion:**

Currently, no randomized controlled trials (RCTs) have been designed to investigate the long-term oncologic outcomes of SILS for CRC. This study is expected to provide clinical evidence of the oncologic outcomes of SILS compared to CLS for CRC to promote its widespread use.

**Trial registration:**

ClinicalTrials.gov: NCT 04527861 (registered on August 27, 2020).

## Background

### Rationale

Colorectal cancer (CRC) is a common malignant tumour of the digestive tract, and its global incidence ranks third among all malignancies [1]. Surgery is a major treatment for CRC. Laparoscopic surgery for CRC has been proven to be safe and effective compared with laparotomy by many randomized controlled trials [2–7], which provides many advantages, including less surgical trauma, better cosmetic results, and a shorter hospital stay [2–7].

Recently, with the extensive development of laparoscopic surgery and the extension of the concept of minimally invasive surgery, many institutions have begun studies on "scarless" surgery to reduce trauma and postoperative pain and improve the cosmetic effect. These studies mainly focused on natural orifice transluminal endoscopic surgery (NOTES) and single-incision laparoscopic surgery (SILS). The current clinical application of NOTES is greatly limited due to difficulties in ethics, gastrointestinal anastomosis, infection prevention, operation platforms, and instrument research [8, 9]. In contrast, SILS is attracting increasing attention. It is a way to transition to “scarless” surgery that is more feasible for generalized use in the future.

Single-incision surgery was reported as early as 1969, when Wheeless et al. [10] performed tubal ligation with laparoscopy through a single surgical channel. In 1997, Navarra et al. [11] first reported transumbilical single-incision cholecystectomy, performed through two 10-mm trocars set in a small transumbilical incision combined with suspension technology. Complete transumbilical cholecystectomy without any additional trocar was first performed by Podolsky et al. in 2007 [12], marking the development of SILS. CRC surgery is generally a semielective surgery. Radical resection is relatively complex and requires a higher degree of proficiency and precision. Therefore, it was not until 2008 that SILS was first reported for colorectal surgery. Bucher et al. [13] and Remzi et al. [14] independently performed right hemicolectomy for benign colonic polyps with good outcomes. In 2009, Bucher et al. [15] successfully performed single-incision laparoscopic radical left colectomy, being the first to apply SILS in malignant colorectal tumours.

Although the number of studies on this new method of surgery has increased year by year, the majority are retrospective studies with small sample sizes, and few are high-quality randomized controlled trials (RCTs). Although the feasibility and short-term safety of SILS were confirmed in most previous studies, the potential benefits are still controversial. Furthermore, the long-term oncologic outcomes remain uncertain, as only a few studies contained long-term survival data [16–18].

To the best of our knowledge, in addition to our single-centre RCT study [19], seven other RCT studies [18, 20–26], including three multicentre studies [18, 23, 25, 26] have been published on SILS for CRC. In the four earlier reported RCT studies [20–22, 24], the sample sizes were calculated inadequately, leading to less reliable conclusions. In addition, in Maggiori et al.’s multicentre study [25], the value of SILS for CRC could not be well assessed due to limited malignant cases. The SIMPLE trial [26] had the largest sample size and multicentre participation, making it the best-designed published RCT study to date. However, similar to Watanabe et al.’s study [18, 23], patients with cancers located in the rectum, descending colon, or transverse colon were excluded. No previous RCT studies were designed with long-term outcomes as the primary study endpoint.

Studies on SILS for CRC are still in the early stage. Therefore, further studies, particularly large-scale, prospective randomized controlled trials, are needed to assess its safety, efficacy, potential benefits, and long-term outcomes to evaluate its value in CRC.

### Objective and hypothesis

This study will investigate the long-term oncologic outcomes of SILS compared to CLS for CRC. The hypothesis is that the oncologic outcomes of SILS for CRC are noninferior to CLS.

## Methods/Design

### Settings

This CSILS study will be a prospective, multicentre, open-label, noninferiority, parallel-group randomized controlled trial. The trial will be conducted at 10 qualified clinical centres in China (Table 1). The CSILS protocol and this manuscript were prepared following the Standard Protocol Items: Recommendations for Interventional Trials (SPIRIT). This manuscript refers to version 1.1 of the full study protocol released on 6 February 2021.

**Table 1:** Participating centers

Centers	Location
Ruijin Hospital, Shanghai Jiao Tong University School of Medicine	Shanghai
Dongfang Hospital Affiliated to Tongji University	Shanghai
Shanghai Cancer Center, Fudan University	Shanghai
RenJi Hospital, Shanghai Jiao Tong University School of Medicine	Shanghai
Changhai Hospital, Navy Medical University	Shanghai
Zhejiang Provincial People's Hospital, People's Hospital of Hangzhou Medical College	Hangzhou, Zhejiang
Shengjing Hospital, China Medical University	Shenyang, Liaoning
Liaoning Cancer Hospital & Institute	Shenyang, Liaoning
Shandong Provincial Hospital Affiliated to Shandong First Medical University	Jinan, Shandong
The General Hospital of Western Theater Command	Chendu, Sichuan

### Eligibility criteria

Patients between 18 and 85 years old, of both sexes, with cT1-4aN0-2M0 CRC diagnosed via computed tomography (CT) and/or colonoscopy according to the 8th Edition of the American Joint Committee on Cancer (AJCC) Cancer Staging Manual, with a tumour located above the peritoneal reflection and a tumour size no larger than 5 cm will be further screened for inclusion. Table 2 shows the detailed inclusion, exclusion, and withdrawal criteria.

**Table 2:** Inclusion, exclusion, and withdrawal criteria

Inclusion criteria	Exclusion criteria	Withdrawal criteria
• 18 years < age < 85 years • Pathological colorectal adenocarcinoma • Tumor located in colon and rectum (the lower border of the tumor is above the peritoneal reflection) • Clinically diagnosed cT1-4aN0-2M0 lesions according to the 8th Edition of AJCC Cancer Staging Manual without preoperative neoadjuvant therapy performed • Tumor size of ≤ 5 cm • Performance status ECOG 0–1 • ASA class I to III • Informed consent	• BMI > 35 kg/m2 • Previous gastrointestinal surgery (except for appendicectomy) • Emergency operation due to complication caused by colorectal cancer (bleeding, perforation or obstruction) • Multiple malignant lesions • Familial adenomatous polyposis (FAP) or Inflammatory bowel disease (IBD) • Requirement of neoadjuvant therapy • Pregnancy or lactation • Severe mental disease • Severe liver and kidney dysfunction, coagulation dysfunction, or with serious comorbidities that cannot perform surgery • Requirement of simultaneous surgery for other disease • Other malignant disease history within 5 years	• Intraoperative or pathological confirmation of invasion of adjacent structures requiring combined organ resection or distant metastasis • Non-colorectal adenocarcinoma confirmed by postoperative pathology • Multiple malignant lesions confirmed by postoperative pathology • Requirement of emergency operation due to the change of illness state • Inability to undergo surgery or anesthesia due to the change of illness state • Receive treatment other than the protocol during the treatment period (except for treatment of tumor recurrence and metastasis) • Patient required to withdraw • Unable to complete the clinical trial due to various reasons • Treatment implemented is proven to violate the study protocol

*BMI* Body mass index, *ASA* The American Society of Anesthesiologists, *ECOG* Eastern Cooperative Oncology Group

Before participating in this study, surgeons will be required to pass a blinded evaluation of their surgical videos. They will be required to provide the CSILS study committee with 3 surgical videos of SILS and 3 of CLS for CRC performed within the last 6 months. The study committee will randomly select 2 surgical videos each and arrange a blind review with 3 peer experts. The surgeon will be permitted to join the study if the 3 reviewers unanimously approve the surgical technique and oncological outcomes shown in the videos.

### Participant timeline and recruitment

**Fig. 1:**
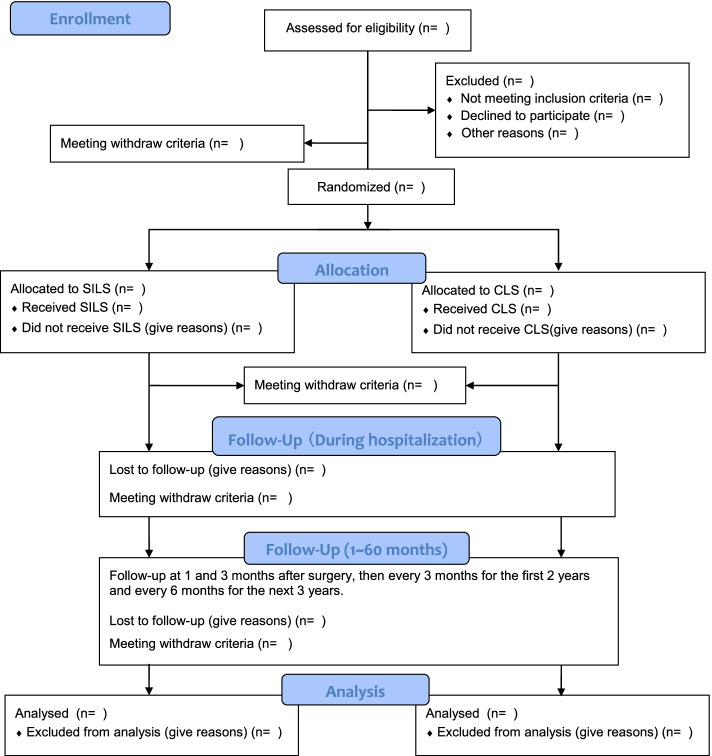
CSILS Flow Diagram

Before the screening, the informed consent form will be verbally explained to all participants. Written informed consent will be acquired from all patients prior to their participation in this study. After enrolment, the baseline characteristics of the participants will be measured preoperatively. Surgery will be performed within 7 days of enrolment. Adjuvant chemotherapy will be considered according to the National Comprehensive Cancer Network (NCCN) guidelines. The first two follow-up visits will be scheduled at one month and three months postoperatively, then every three months for the first two years and every six months for the next three years. This trial is scheduled to last for 96 months and the recruitment started on April 8, 2021. Recruitment advertisements for the study will be posted on notice boards in participating centres. The CSILS flowchart was designed based on consolidated standards of reporting trials (CONSORT 2010 Flow Diagram, Fig. 1). The study schedule is shown in Table 3.

**Table 3:**
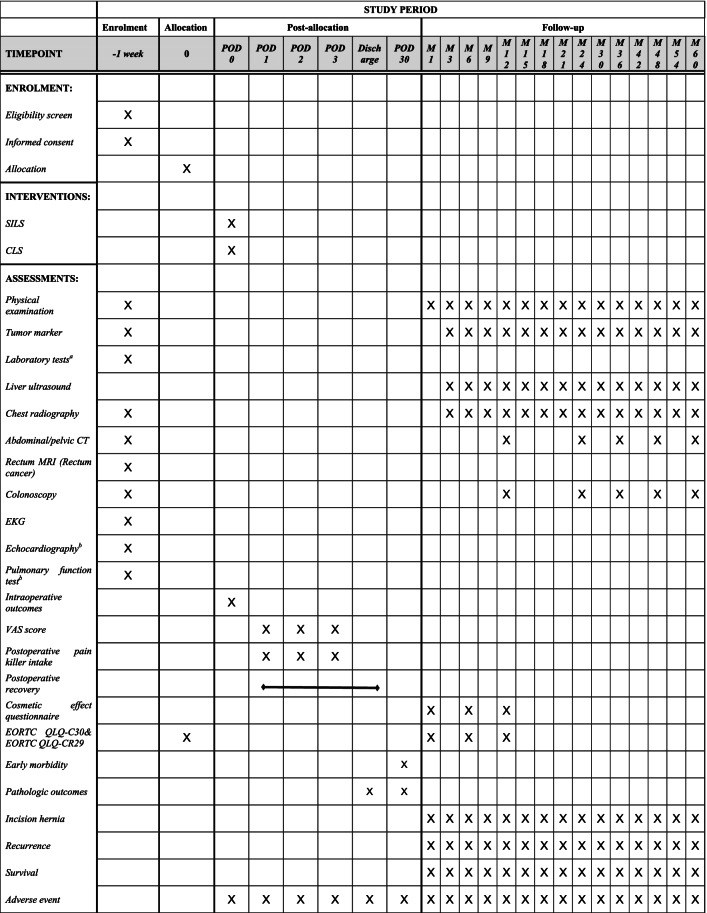
Study schedule

*POD* Postoperative day, *M* Month, *SILS* Single-incision laparoscopic surgery, *CLS* Conventional laparoscopic surgery, *VAS* Visual analogue scale, *CT* Computed tomography, *MRI* Magnetic Resonance Imaging, *EKG* Electrocardiogram, *QLQ-C30* Quality of Life Questionnaire-Core 30, *QLQ-CR29* Quality of Life Questionnaire-Colorectal 29, *EORCT* European Organization for Research and Treatment of Cancer

^a^ Laboratory tests include routine blood tests, liver and kidney function tests, electrolytes tests, and coagulation tests

^b^ These examinations are optional depending on the patient's condition

### Interventions

Preoperative nutritional support, bowel preparation, prophylactic systemic antibiotics, prophylactic anticoagulation, respiratory function training, and prophylaxis for other potential high-risk complications are administered according to the patient's condition and the routine of each study centre. Nasogastric tubes will not be routinely used.

Radical resection (R0) based on the same oncological principles is required for all operations. The principles include adequate surgical resection margins, complete or total mesocolic excision (CME/TME), and D3 lymph node dissection. Intraperitoneal operations must be performed using laparoscopic instruments supported by a camera system. In the SILS group, the operations will be performed through a single incision, usually with a transumbilical approach. A single port device and laparoscopic instruments will be placed through the small incision. In the CLS group, the operation will be performed with 3 to 5 trocars. Conventional laparoscopic instruments, precurved instruments, articulating instruments, 3D laparoscopes, and flexible laparoscopes, among others., may be used during the operation. Intestinal segment mobilization, lymph node dissection, and vascular ligation should be performed under laparoscopy. The specimen excision and the anastomosis will be allowed to be performed in an open state using an auxiliary small incision (usually the same size as the tumour). Auxiliary incisions will generally be limited to one, except in cases where a stoma is needed. According to the site of the tumour, appropriate surgical methods, including right colectomy, left colectomy, sigmoidectomy, and anterior resection, will be selected. Prophylactic ileostomy will be performed when necessary. Placement of drainage tubes will be decided by the surgeons. Unedited video recordings of the laparoscopic operations, photographs of the incisions, and photographs of the specimens will be documented.

The treatment strategy will be required to change (SILS to additional trocar/laparotomy; CLS to laparotomy) if one of the following happens: intractable intra-abdominal haemorrhage, severe organ damage, other serious or life-threatening complications related to surgical procedures or carbon dioxide pneumoperitoneum, or other technical or instrumental factors requiring conversions. When the length of the auxiliary incision is larger than 10 cm, conversion to laparotomy will be considered.

### Primary endpoint

The primary endpoint is 3-year disease-free survival (DFS) measured at 36 months postoperatively. DFS will be calculated from the time of surgery to the time of tumour recurrence or death from tumour reasons (in the case of an unknown tumour recurrence date). If neither death nor tumour recurrence is observed during the follow-up, the final date of confirmation of disease-free survival will be marked as the date of the latest outpatient visit or the examination received. Recurrence will be confirmed by radiological or histological methods.

### Secondary endpoint

The secondary endpoints include intraoperative outcomes, postoperative recovery, postoperative pain assessment, pathological outcomes, early morbidity and mortality rate, cosmetic effects, quality of life, 3-year overall survival (OS), incidence of incisional hernia, 5-year DFS and 5-year OS. The intraoperative outcomes include operative time, estimated blood loss, complications, incision length, total incision length, and conversion rate measured intraoperatively. The postoperative recovery outcomes include time to first flatus/stool, time to liquid diet and soft diet, and length of theoretical hospital stay measured upon daily visits during hospitalization. To adjust for the potential impact of the nonmedical variables of different centres, a theoretical length of hospital stay will be used, defined as the postoperative day when patients meet the following predefined discharge criteria: no fever; no need for intravenous nutritional support; passage of first flatus/stool; able to leave the bed with limited assistance. The postoperative pain assessment will be measured on postoperative Days 1, 2, and 3, including the postoperative pain score, which will be recorded using the visual analogue scale (VAS) pain score and dosage of postoperative pain killer intake. The pathologic outcomes, including tumour size, number of harvested lymph nodes, proximal and distal resection margins, and mesangial integrity evaluation (rectum), will be determined postoperatively by pathological examination. Early morbidity, graded according to the Clavien–Dindo classification is defined as postoperative complications observed within 30 days after surgery. The cosmetic effect will be measured by a questionnaire at 1, 6, and 12 months postoperatively. Quality of life will be measured using the European Organization for Research and Treatment of Cancer (EORTC) Quality of Life Questionnaire-Core 30 (QLQ-C30) and the EORTC Quality of Life Questionnaire-Colorectal 29 (QLQ-CR29) preoperatively and at 1, 6, and 12 months postoperatively. The incidence of incisional hernia will be measured at 60 months postoperatively. Incisional hernia will be confirmed by physical examination or radiological methods. Other long-term outcomes include 3-year overall survival (OS), 5-year DFS, and 5-year OS.

### Randomization and blinding

This will be an open-label trial. Surgical procedures and postoperative intervention will not be concealed from patients or investigators. The participants will be randomly assigned to either the CLS or SILS group at a 1:1 ratio using a central, dynamic, and stratified block randomization method. The control factors include age, preoperative stage, and planned surgical intervention. An investigator from each centre will fill in the information of every participant through the network, and the central random system will analyse the information and give the random number and grouping.

### Data management

An electronic data capture (EDC) system designed by the Clinical Research Center, Shanghai Jiao Tong University School of Medicine, will be used for data collection and query handling. The investigators will log into the EDC system via the website and record the data on the electronic case report form (eCRF). The investigators are required to submit the data within 7 days of its generation to ensure the timeliness of the data recorded. The clinical research associates are responsible for verifying the raw data and the eCRF to ensure the authenticity, accuracy and completeness of the data recorded.

### Data monitoring

An Independent Data Monitoring Committee (IDMC) composed of an independent contract research organization (CRO) will be set up to ensure the protection of patients, to ensure the ethical conduct of the study, to ensure the accuracy, completeness, and timeliness of the data, to evaluate the benefit/risk ratio of the study and to ensure an independent review of the scientific outcomes during and at the completion of the study. The committee is independent from the sponsor and has no competing interests. Monitoring will be conducted at each centre on a monthly basis during the enrolment period and every two months during the follow-up period.

### Sample size calculation

In this study, the 3-year DFS is the main effectiveness evaluation index for noninferiority evaluation. According to previous studies [27–29], the 3-year DFS of the CLS group was approximately 75%. This study assumes that the 3-year DFS of the study group is the same as that of the control group. The expected enrolment time is 3 years, and the follow-up time to reach the primary endpoint is 3 years. If the acceptable noninferiority limit of DFS in the three years of this study is 10%, when DFS is exponentially distributed, the corresponding HR limit is 1.49. This number will be combined with "Log-Rank Tests for Noninferiority" in the professional sample size estimation software NCSS-PASS (14th edition, NCSS, LLC, Utah, USA). When the unilateral statistical significance level is 0.025, the inspection efficiency is 80%, and the study group and the control group will be allocated in the ratio of 1:1, the required sample size of each group will be 284. Considering that the largest abscission rate in this clinical study is approximately 20%, it was finally determined that the sample size of each group will be 355 cases, and the total number of cases required will be 710.

### Statistical methods

SAS 9.4 statistical software will be used to perform the statistical analyses. The noninferiority analysis for the primary endpoint of 3-year DFS will be conducted by comparing 95% confidence intervals of survival rates between the test and control groups on a modified intent-to-treat (MITT) population basis. Baseline data and validity analyses will be conducted on a modified intent-to-treat (MITT) basis. The primary endpoint will also be analysed on a per-protocol (PP) basis, with the MITT analysis results prevailing. Safety evaluation, including laboratory test data, will be conducted in the safety analysis population (SAP). There is no planned interim analysis for the 3-year DFS rate. Continuous data will be described as the means with standard deviations or medians with interquartile ranges, and categorical data will be expressed as frequencies and percentages. Comparisons between groups will be conducted using the Wilcoxon rank-sum test, Student’s t test, Pearson χ2 test, and Fisher exact test, as appropriate. Survival data will be analysed using the Kaplan–Meier method and log-rank test. A general linear model for quantitative indicators, logistic regression for qualitative indicators, and Cox's proportional hazards model for survival data will be used to assess the effects of baseline, treatment, centre, and treatment-by-centre interactions. A two-sided *p* value < 0.05 will be considered statistically significant.

### Adverse events

Based on the qualifications of the participating centres and surgeons in this study, treatment-related deaths or life-threatening complications due to surgery are generally unlikely to occur. All adverse events (AEs) will be graded according to the Clavien–Dindo classification or Common Terminology Criteria for Adverse Events (CTCAE) version 5.0 and recorded. Any death or unexpected Grade 4 adverse event will be reported urgently to the principal investigator within 24 h. It will also be reported to the Ethics Committee within 72 h, and a detailed medical report will be generated within 15 days. The CSILS Efficacy and Safety Evaluation Committee will be responsible for assessing the quality of surgery by reviewing surgical videos and monitoring each center’s safety. If treatment-related deaths or life-threatening complications occur in up to 3% of the preallocated proportion of patients at each centre, patient enrolment will be terminated immediately and the Ethics Committee will reassess the safety of the trial.

## Discussion

As an emerging minimally invasive approach, SILS is considered the next major advance in minimally invasive surgical approaches for CRC treatment [30]. However, its use is limited by technical challenges, including loss of triangulation, in-line orientation, and instrument collision, among others, as well as inconclusive long-term oncological outcomes. The skill sets and ergonomic demands of this new method of surgery cannot be directly adapted from CLS experience [31]. Therefore, to ensure the quality of the surgery, surgeons participating in this study must pass a blind review of the surgical videos.

Patients with tumours below the peritoneal reflection will be excluded, as our prior surgical experience showed that SILS treatment is very difficult to complete for these patients and often requires conversions intraoperatively. The slim and narrow pelvic space makes it difficult to expose the surgical field and the surgical instruments collide frequently, resulting in poor mobilization of the low rectum [32]. Jung et al. [33] reported that in 144 SILS low anterior resection (LAR) cases and 3 SILS abdominoperineal resection (APR) cases, an additional trocar was added in 107 patients. They consider SILS plus one additional trocar a second-string procedure during SILS LAR because of its unique complexity and difficulty in achieving distal division with an insufficiently angled stapler and without proper total mesorectal excision. Patients with neoadjuvant treatment will also be excluded, as these patients often develop tissue oedema and fibrosis, making SILS surgery more difficult.

Currently, no RCT has been designed to investigate the long-term oncologic outcomes of SILS for CRC. In addition, it remains controversial whether SILS has the potential benefits of better cosmetic effects and less postoperative pain and whether SILS increases the incidence of incisional hernias. This study is expected to provide relevant high-quality clinical evidence, particularly of the oncologic outcomes of SILS compared to CLS for CRC, and promote its widespread use.

## Data Availability

The datasets generated during the current study will be available from the CSILS Research Committee on reasonable request after the publication of the main findings.

## Abbreviations

AE: Adverse events
AJCC: American Joint Committee on Cancer
APR: Abdominoperineal resection
ASA: The American Society of Anaesthesiologists
BMI: Body mass index
CLS: Conventional laparoscopic surgery
CME: Complete mesocolic excision
CRC: Colorectal cancer
CT: Computed tomography
CTCAE: Common Terminology Criteria for Adverse Events
CRO: Contract research organizations
DFS: Disease-free survival
eCRF: Electronic case report form
ECOG: Eastern Cooperative Oncology Group
EDC: Electronic data capture
EKG: Electrocardiogram
EORTC: European Organization for Research and Treatment of Cancer
IDMC: Independent Data Monitoring Committee
IEC: Independent Ethics Committee
LAR: Low anterior resection
MITT: Modified intent-to-treat
MRI: Magnetic Resonance Imaging
NCCN: National Comprehensive Cancer Network
NOTES: Natural orifice transluminal endoscopic surgery
OS: Overall survival
POD: Postoperative day
PP: Per-protocol
RCT: Randomized controlled trials
SAP: Safety analysis population
SILS: Single-incision laparoscopic surgery
TME: Total mesorectal excision
VAS: Visual analogue scale
